# Medical students and COVID-19: lessons learnt from the 2020 pandemic

**DOI:** 10.1136/postgradmedj-2020-138559

**Published:** 2020-08-11

**Authors:** Marta de Andres Crespo, Henry Claireaux, Ashok Inderraj Handa

**Affiliations:** Medical Sciences Division, Oxford University, Oxford, UK; University of Oxford Nuffield Department of Orthopaedics Rheumatology and Musculoskeletal Sciences, Oxford, UK; Nuffield Department of Surgery, Oxford, UK

At the start of 2020, a medical student’s largest problem was passing their next exam at medical school. To doctors, these students were people who needed to be taught but were of minimal help in the hospital. The COVID-19 pandemic changed this for both parties. There are some crucial lessons to be learned.

As COVID-19 started to overwhelm the National Health Service (NHS), final-year medical students saw exams cancelled, graduations accelerated and their career start date fast-forwarded. With this came student anxieties regarding their preparedness for the job and their fear of being ill-equipped. Starting as a doctor is a difficult transition under normal circumstances, but it is even tougher in a global pandemic. As these anxieties grew, some students wrote letters to publicise their concerns. One such letter was written relatively early on and published in the *Lancet*.^1^ It asked for greater guidance and transparency regarding indemnity, contracts, expected roles and responsibilities of students. As a result of this, a survey was conducted with Oxford University medical students to give students a voice and understand their involvement in the current pandemic. Many people experienced a significant degree of anxiety during the past 3 months and medical students are no exception. There are some key lessons to learn from the results of this survey to better equip this subset of the population in future, either for a second wave of COVID-19 or a different infectious pandemic altogether. It is inevitable that another pandemic will occur. The only questions are when, and if we will be better prepared for it next time.

## LESSON 1: MEDICAL STUDENTS PLAYED CRUCIAL ROLES IN HOSPITALS, PRIMARY CARE AND IN COMMUNITIES

The vast majority (80%) of medical student respondents were working across NHS Trust hospitals, in primary care and in the community, with a further 11% still waiting to be tasked.

In hospitals, students were placed in Accident and Emergency, triaging patients into either COVID-19-infected or non-infected groups. In the intensive care unit, they played key liaison roles with families who were not able to visit their loved ones. They also worked in hospitals in roles similar to foundation year doctors, taking bloods and placing cannulas. Others worked in hospital pharmacies delivering medications to the wards or in PCR labs carrying out testing of COVID-19.

In primary care, students routinely telephoned elderly people to check on their welfare. They discussed important issues, including end-of-life considerations with patients. A daily teleconference with a doctor was used to discuss cases, carry out teaching on communication skills, develop primary care research skills and manage the students’ own mental well-being. Lastly, in communities, students helped deliver groceries to those who had to isolate through various organisations. They also had managerial roles to organise community efforts and maximise efficiency. Several students remarked on the differences in volunteering effort between Oxford and more rural areas where their friends and families lived, giving examples of grandparents struggling to cope with less community support schemes in operation.

From this, we are able to see that medical students played a vital role in the pandemic effort. They occupy a unique position in our society in that they are not laypeople but neither are they doctors so they are able to bridge the gap between the two. They are an extra pair of hands that can improve the efficiency of hospitals and free up more senior doctors for the management of complex cases.

## LESSON 2: CONTINUING MEDICAL EDUCATION ONLINE

One of the most significant impacts of COVID-19 on medical students was that the medical school closed on March 13, 2020 and, along with it, the teaching it provided. Despite this, 71% of students were working on matters related to their degree, many of which were doing so alongside volunteering roles. Preclinical students had exams to revise for but clinical years had little guidance of what was expected. They turned to self-directed learning using textbooks and online resources.

Oxford medical school has had to update its online resources extensively as all lectures for current 5th-year undergraduates have been cancelled and moved wholly online, with specific online modules to cover each topic. Medicine is one of the few degrees for which it is impossible to teach without any in-person contact. The vast majority of learning in clinical years depends on clinical placements and interactions with doctors and patients alike. However, it still involves building a mental model and pattern recognition, both of which can be learnt from the comfort of your own home. In an age where computers and technology are becoming increasingly prevalent in our lives, investing in online platforms and developing resources to teach the degree virtually, guarantees a continuation of teaching, even during a pandemic.

## LESSON 3: THE IMPACT OF COVID-19 ON MEDICAL STUDENT WELL-BEING AND HOW TO MANAGE THIS

In our survey, 65% of respondents reported that COVID-19 had negatively impacted their mental well-being. Their concerns surrounded three key areas, the most significant of which was the risk of infection to others. Within Oxford, there was a heavy emphasis on following personal protective equipment (PPE) guidance and social distancing and yet students still felt unprepared. On a larger scale, from the news-reported nationwide PPE shortages, one can assume that not all medical schools and their students were as fortunate as those participating in this survey. The second main concern surrounded the impact of the pandemic on their education and ability to progress to the next year. This has already been addressed in Lesson 2. Finally, students, much like the general public, were particularly anxious about the uncertainty regarding the duration of social distancing measures and general isolation policy. Students too had lost loved ones in the pandemic and were unable to visit them. Despite the increased anxiety and poor mental health levels, social isolation measures meant that there was a reduction in access to help, with many students having to cope on their own.

General coping strategies involved exercise (92%), keeping in touch with family and friends (92%) and music (67%), be it playing an instrument or simply listening to it. There were specific suggestions for medical school support, some of which have since been implemented by the Oxford medical school. First, 40% of students asked for more regular updates from the medical school, with three students suggesting a weekly newsletter. At the start of the pandemic, the medical school only updated its students when there was news to tell. In this survey, it was clear that students preferred being kept informed on a regular basis, even if little had changed. Since this survey was completed, Oxford medical school has indeed sent weekly emails on Friday afternoons to its students, explaining the next steps they are conducting in starting clinical placements and new online resources they have created. The survey respondents also suggested releasing a survey to students regarding their well-being and take a proactive approach to the problem. Though this was not done, the Oxford medical school created online ‘drop-in’ sessions that students could book if they felt they needed further support with their mental health.

## Conclusion

A recent systematic review conducted by Ashcroft *et al* investigated the value of implementing a disaster training programme for medical students.^2^ They found that these improved preparedness, knowledge and skills that could be of use to medical students if another pandemic occurs and they are recruited to assist. This pandemic training programme could target some of the key lessons learnt from this survey. First, it could train medical students on managing emergency situations and conducting tasks in the hospital, primary care or in communities while minimising the risk of infection to themselves. Second, it could teach them to recognise burnout or anxiety as a result of a highly strenuous medical situation such as a pandemic and give them the tools to manage this when normal mental health resources are not open. Last, it would give students a platform to voice their concerns and have them addressed.

Thankfully, the rates of COVID-19 infection are now on the decline and it appears the first wave of this pandemic is coming to an end. However, the threat of a potential second wave still exists and so it is of the utmost importance that we learn from the past few months. Medical student assistance has occurred across the country and it is highly plausible that more medical students will be needed in a future pandemic. Therefore, we should take the time we have now to re-assess and take note of the lessons learnt from the past 3 months.

**Main messages**

1. Medical students have played a variety of roles in the COVID-19 pandemic, working amongst healthcare professionals in hospitals, primary care and in communities.
2. A significant proportion of students were still awaiting jobs when completing this survey. Thus, there is a lot of potential for the further use of medical students in future pandemics, if they are properly trained and incorporated into healthcare teams.
3. Medical student learning should be adapted to include more online modules so that in future, if medical schools close, medical education does not need to come to a halt.
4. Mental wellbeing is an important aspect of medical student health that should be investigated and addressed as the majority of students have found their mental health to be negatively impacted by the pandemic. Support measures should be put in place to help students with both the fears of the pandemic itself and the measures taken to combat it. Further investigation into this subset of the population is needed.
